# Observations in Regard to the Application of Carbolic Acid

**Published:** 1896-06

**Authors:** 


### ABSTRACTS FROM FOREIGN JOURNALS.[1]

Observations in Regard to the Application of Carbolic Acid. Prof. J. Rosenbach (Göttingen), in the medical periodical *Die Praxis*, reports a number of cases in which burning (mortification) set in a few hours after the application of a weak carbolic solution (the carbolic water sold by apothecaries, containing about 3 per cent.) to bandages. In most cases it occurred upon the fingers, which were lost in this way; weakly individuals, *i.e.*, women and children, are most liable to this danger. For this reason it has been proposed to forbid the dispensing of carbolic solutions without a physician's prescription. Rosenbach considers that such measures would be still more effective if, besides physicians, the members of the various sanitary boards were informed of the danger of carbolic gangrene.—*Thierärzt. Centralblatt*, Jan. 1, 1896.

[1] Under the general direction of J. Preston Hoskins, Princeton, N. J.

